# Galvanic Vestibular Stimulation Improves Subnetwork Interactions in Parkinson's Disease

**DOI:** 10.1155/2021/6632394

**Published:** 2021-05-13

**Authors:** Aiping Liu, Huiling Bi, Yu Li, Soojin Lee, Jiayue Cai, Taomian Mi, Saurabh Garg, Jowon L. Kim, Maria Zhu, Xun Chen, Z. Jane Wang, Martin J. McKeown

**Affiliations:** ^1^School of Information Science and Technology, University of Science and Technology of China, Hefei, China; ^2^Wellcome Centre for Integrative Neuroimaging, University of Oxford, Oxford, UK; ^3^Pacific Parkinson's Research Centre, Vancouver, Canada; ^4^Department of Neurology, Neurobiology and Geriatrics, Xuanwu Hospital of Capital Medical University, Beijing Institute of Brain Disorders, Beijing, China; ^5^Epilepsy Centre, Department of Neurosurgery, The First Affiliated Hospital of USTC, Division of Life Sciences and Medicine, University of Science and Technology of China, Hefei, China; ^6^Department of Electrical and Computer Engineering, University of British Columbia, Vancouver, Canada; ^7^Department of Medicine (Neurology), University of British Columbia, Vancouver, Canada

## Abstract

**Background:**

Activating vestibular afferents via galvanic vestibular stimulation (GVS) has been recently shown to have a number of complex motor effects in Parkinson's disease (PD), but the basis of these improvements is unclear. The evaluation of network-level connectivity changes may provide us with greater insights into the mechanisms of GVS efficacy.

**Objective:**

To test the effects of different GVS stimuli on brain subnetwork interactions in both health control (HC) and PD groups using fMRI.

**Methods:**

FMRI data were collected for all participants at baseline (resting state) and under noisy, 1 Hz sinusoidal, and 70-200 Hz multisine GVS. All stimuli were given below sensory threshold, blinding subjects to stimulation. The subnetworks of 15 healthy controls and 27 PD subjects (on medication) were identified in their native space, and their subnetwork interactions were estimated by nonnegative canonical correlation analysis. We then determined if the inferred subnetwork interaction changes were affected by disease and stimulus type and if the stimulus-dependent GVS effects were influenced by demographic features.

**Results:**

At baseline, interactions with the visual-cerebellar network were significantly decreased in the PD group. Sinusoidal and multisine GVS improved (i.e., made values approaching those seen in HC) subnetwork interactions more effectively than noisy GVS stimuli overall. Worsening disease severity, apathy, depression, impaired cognitive function, and increasing age all limited the beneficial effects of GVS.

**Conclusions:**

Vestibular stimulation has widespread system-level brain influences and can improve subnetwork interactions in PD in a stimulus-dependent manner, with the magnitude of such effects associating with demographics and disease status.

## 1. Introduction

Parkinson's disease (PD) is characterized by motor symptoms of rigidity, tremor, bradykinesia, and nonmotor symptoms such as affective disorders and cognitive decline. While dopaminergic treatments benefit all PD patients, nonmotor and nondopaminergic disabilities, such as those related to balance, are not satisfactorily controlled with current medication [1]. Deep brain stimulation (DBS) is a highly effective treatment to alleviate motor symptoms such as stiffness, slowness, and tremor. While DBS suppresses abnormal neuronal activity around the stimulating sites [2], it also rebalances whole-brain dynamics by shifting global integration and synchronization towards a regime seen in healthy subjects [3]. As DBS is inherently invasive and expensive and has risks associated with the procedure, there is interest in noninvasive brain stimulation techniques to modulate pathological brain activities [4].

Galvanic Vestibular Stimulation (GVS) is a safe and potential portable brain stimulation technique to noninvasively activate vestibular afferents by applying weak electrical currents to the mastoid processes behind the ears. It ultimately influences downstream activity in both the vestibular network and regions associated with multisensory processing. There is growing evidence demonstrating beneficial GVS effects in PD such as improving overall motor deficits [5, 6], manual tracking performance [7], balance [8], connectivity to the pedunculopontine nucleus (PPN) [9], and deficient interhemispheric connectivity [10]. For a comprehensive review on GVS in the treatment of PD, we refer the reader to [11].

However, comprehensive evaluation of noninvasive brain stimulation in PD is still limited. A key question is, what do biomarkers need to be optimized with brain stimulation? Brain stimulation methods are typically designed to augment compensatory functions while alleviating maladaptive changes, a process that may need to be individualized [4]. For example, while GVS is known to activate vestibular afferents to the thalamus as well as the basal ganglia [12], how this translates into improved motor performance is incompletely known. Kim et al. have shown that GVS modulates brain synchrony patterns [13]; however, customization of stimulus parameters or suggesting new stimulus patterns will likely require optimization of a quantitative feature. Since focally applied DBS, a well-accepted treatment of PD, results in a shift overall brain dynamics in PD [3], here we investigate system-level effects of GVS in PD.

There is increasing recognition of the role of interacting brain subnetworks, something that may provide a candidate biomarker for stimulus effects, during normal brain functioning and in disease states [14]. The brain can be considered as a system of interconnected subnetworks designed to decrease wiring cost while still maintaining efficiency of information transmission between distant brain regions. Neurodegenerative disorders may target network-level abnormalities [15]. For example, dysfunction of default mode network (DMN) connectivity has been associated with cognitive decline in PD [16]. Therefore, here we are interested in studying the influence of different GVS stimuli on subnetwork interactions.

A number of methods have been proposed to study subnetwork interactions in fMRI. Group-level Independent Component Analysis (ICA), which finds spatially independent component maps, can be used to explore network interactions by considering each component map as a spatially distributed network. Therefore, examining the associations between the corresponding time courses of each component provides a way to assess network interactions [17]. With a moving-window approach, time-varying interactions can also be assessed [18]. Causal relationships between brain activation networks can be examined by testing for Granger causality between component time courses [19]. However, group ICA approach requires that the data be spatially normalized to a common template space. In studies involving subjects with neurodegenerative disorders, where focal and heterogeneous atrophy may occur, the effects of this normalization step, particularly on the basal ganglia, vital for assessment of diseases like PD, are unclear. An alternative is to perform analysis at the Region of Interest (ROI) level, making the much less stringent assumption that the ROIs, as opposed to the individual voxels, correspond across subjects. By correlating the averaged signals within ROIs, a network can be created, which can then be subdivided into subnetworks using the technique of modularity detection, which defines subnetwork boundaries by jointly maximizing the connections within subnetworks and minimizing the connections between subnetworks. Detection between subnetworks can then be estimated with canonical correlation analysis (CCA), a powerful tool to examine the linear relationships between two sets of variables, which has been widely used for multimodality data fusion in neuroimaging analysis [20, 21]. In the current context, CCA would determine the weightings across different ROIs defining a subnetwork, so that the weighted sums from two subnetworks are maximally correlated [22]. Typically, the weight vectors estimated by CCA contain both positive and negative values, which may represent the mixture of synchronization and desynchronization between ROIs within the subnetworks. As we prefer to investigate the synchronized activities between subnetworks, a nonnegativity constraint is preferable.

In this paper, we investigate the interactions between subnetworks with nonnegative CCA and evaluate the effects of different GVS stimuli in subjects with PD. In addition, we show that GVS improves subnetwork interactions as a function of stimuli characteristics and demographics. This study has been partially presented as a poster in the “3rd International Brain Stimulation Conference” [23].

## 2. Materials and Methods

### 2.1. Subjects

Thirty-two subjects with PD (10 females; age: 68.03 ± 5.85; UPDRS III: 26.5 ± 10.39; Hoehn and Yahr scale: 2.03 ± 0.74) and fifteen age-matched healthy controls (HC, 5 females; age: 69.4 ± 4.76) were recruited from Pacific Parkinson's Research Center (PPRC) at the University of British Columbia (UBC). There was no significant difference in sex distribution and age between PD and HC subjects. Because we were interested in the complementary effects of GVS, PD subjects were clinically assessed in the on-medication state before the experiment. All patients had mild-to-moderate PD (Hoehn and Yahr stages I–III). Most of the PD subjects had mild tremor and rigidity, and none of them presented freezing of gait. The detailed demographic data are provided in Table 1 and the subscores of tremor, rigidity, bradykinesia, and gait/posture are further described in Table 2. The study was approved by the UBC Ethics Review Board and all the participants provided written, informed consent prior to the experiment.

### 2.2. Galvanic Vestibular Stimulation

Digital signals of the GVS stimuli were first generated on a PC with MATLAB (MathWorks, MA, USA) and were converted to analog signals via an NI USB-6221 BNC digital acquisition module (National Instruments, TX, USA). The analog command voltage signals were then subsequently passed to a bipolar, constant current stimulator (DS5 model, Digitimer Ltd., U.K.). The DS5 constant current stimulator was isolated in the console room with the output cable leading into the scanning room through a waveguide. Along the twisted coaxial output cable, four inductance capacity filters spaced 20 cm apart and tuned for the Larmor frequency (128 MHz) were custom-built. Near the subject, high-resistance radio translucent carbon-fiber leads (Biopac Inc., Montreal, Canada) were connected to pregelled Ag/AgCl electrodes that were MR-compatible (Biopac Inc., Montreal, Canada). For bilateral stimulation, an electrode was placed over the mastoid process behind each ear. Since the GVS stimuli are alternating current (AC), the anode and cathode are not fixed on one side (as for DC) but they are alternating. Each subject had one fMRI scan containing four GVS conditions. The order of GVS condition was kept consistent to be sham (Rest), noisy GVS (GVS1), sinusoidal GVS (GVS2), and 70-200 Hz multisine GVS (GVS3) across all the participants. The noisy stimulus was zero-mean with 1/f-type power spectrum between 0.1 and 10 Hz and the sinusoidal stimulus was a 1 Hz sine wave. For the multisine stimulus, the frequencies were uniformly distributed every 0.4 Hz in the 70-200 Hz band (i.e., 70, 70.4, 70.8,…, 200 Hz) and the phases were chosen by a clipping algorithm to minimize the crest factor [24]. To avoid poststimulation effects, we allowed a 2-minute break between the GVS conditions. To the best of our knowledge, after-effects of GVS on cortical activation have not yet been fully investigated. However, we believe that the break time was sufficient based on literature on the after-effects of transcranial alternating current stimulation [25].

Since individuals have an inherently subjective perception of GVS, prior to scanning, we determined the individual sensory threshold level (cutaneous sensation at the electrode site) utilizing systematic procedures that we and others have used in prior GVS studies [7–9, 26–28]. We delivered GVS at 90% of the individual threshold level.

### 2.3. fMRI Data

A 3T scanner (Philips Achieva 3.0 T R3.2; Philips Medical Systems, Netherlands) equipped with a head coil was used to obtain the resting state imaging data. Before scanning, all the subjects were instructed to lie on their back in the scanner and have several minutes to acclimatize themselves to the scanner environment with eyes closed. High-resolution T1-weighted anatomical images were acquired with repetition time of 7.9 ms, echo time of 3.5 ms, and flip angle of 8°. Blood oxygenation level-dependent (BOLD) contrast echo-planar (EPI) T2*∗*-weighted images were acquired with the following specifications: repetition time of 1985 ms, echo time of 37 ms, flip angle of 90°, field of view of 240 mm × 240 mm, matrix size of 128×128, pixel size of 1.9 mm × 1.9 mm, and the scanning time of 8 mins for rest condition and 5 mins for GVS conditions, respectively.

### 2.4. Preprocessing and ROI Selection

Preprocessing of fMRI data was accomplished using AFNI procedures for despiking, slice timing correction, and 3D isotropic reslicing, as well as movement correction for any major head movements during the scan using rigid body alignment. FreeSurfer was used to perform the standard brain parcellation on T1-weighted images, and coregistration was performed between functional data and parcellated structural images in FSL. In this study, rather than transforming all fMRI data to a common template, all analysis was done in the individual fMRI space, which prevents introducing any unwanted distortions in the fMRI data by registering it to a common template. Nuisance time courses were voxel-wise regressed from the processed data to remove sources of variance such as head-motion parameters, their temporal derivatives, and their squares; white-matter signal; and CSF signal. Then fMRI signal was detrended by removing any linear or quadratic trends. The fMRI data were finally spatially smoothed by a 6 × 6 × 6 FWHM Gaussian kernel and bandpass filtered at 0.01 Hz to 0.08 Hz as recommended. 76 ROIs were chosen in this study as shown in Table 3, which include the representative regions from visual, motor, sensory, attentional, cerebellar, and basal ganglia areas.

### 2.5. Connectivity Network Estimation and Subnetwork Identification

For each subject, we applied error-rate-controlled Bayesian network learning approach, PCfdr, to estimate the interactions between ROIs [29]. The PC algorithm, named after the authors [30], tests for conditional dependence/independence relationships between variables in a computationally efficient and asymptotically reliable manner [30]. The PCfdr algorithm, which integrates a false discovery rate (FDR) control procedure into the original PC algorithm, results in an FDR controlled binary undirected connectivity network [29]. The significance level of FDR in this study was set to be 0.05.

Based on the ROI-level connectivity networks estimated with the PCfdr algorithm, the optimal nonoverlapping subnetworks were identified for each subject by modularity optimization, which maximizes the connections within subnetworks while minimizing the connections between subnetworks [31, 32]:

(1) $$\begin{array}{c} Q=\frac{1}{2m}\;\underset{ij}{\sum }\left[a_{ij}-\frac{k_{i}k_{j}}{2m}\right]\delta \left(\sigma_{i},\sigma_{j}\right), \end{array}$$

where *a*_*ij*_ represents the number of connections between node *i* and node *j* and *k*_*i*_=∑_*j*_*a*_*ij*_ denotes the node degree. *δ*(*σ*_*i*_,  *σ*_*j*_) is the Kronecker delta function, which is equal to one when node *i* and node *j* are assigned to the same module and zero otherwise. To further control the number of communities estimated by modularity maximization, a resolution parameter *γ* can be integrated as [33]

(2) $$\begin{array}{c} Q=\;\frac{1}{2m}\;\underset{ij}{\sum }\left[a_{ij}-\gamma \frac{k_{i}k_{j}}{2m}\right]\delta \left(\sigma_{i},\sigma_{j}\right). \end{array}$$

By varying *γ*, we can control the resolution of the identified modules and thus the number of the modules.

A representative community structure was estimated using consensus clustering by combining partitions of all the subjects [34]. We generate a new set of similarity relationships between ROIs according to their clustering similarity at the subject level, and modularity maximization was applied to obtain the group representative subnetwork structures. The details of the consensus clustering can be found in Supplementary Appendix A.

### 2.6. Subnetwork Interaction

To investigate the subnetwork interactions, nonnegative canonical correlation analysis (nCCA) was adopted to estimate the associations between subnetworks. Suppose that *X*=(*x*_1_, *x*_2_,…,  *x*_*n*_) ∈ *R*^*T∗n*^ represents fMRI signals from one subnetwork with sample length as *T* and the number of ROIs as *n*. *Y*=(*y*_1_, *y*_2_,…, *y*_*m*_) ∈ *R*^*T∗m*^ represents the fMRI data from another subnetwork with *m* ROIs. CCA finds the linear combinations of *X* and *Y*, which achieve the maximum correlation with each other. It can be formulated as the optimization problem:

(3) $$\begin{array}{c} \underset{w,v}{\max}\;w^{\prime }X^{\prime }Yv,\\ s.\;t.\;w^{\prime }X^{\prime }Xw=1,\;v^{\prime }Y^{\prime }Yv=1,\; \end{array}$$

where *w* and *v* are the weight vectors for *X* and *Y*, respectively. The weighted time courses *Xw* and *Yv* can be considered to be representative signals of the respective subnetworks. However, the weight vectors contain both positive and negative values, mixing the synchronization and desynchronization between ROIs within the subnetworks. As we prefer to investigate the synchronized activities between subnetworks, nonnegativity constraints are imposed as *w* ≥ 0 and *v* ≥ 0 [35].

To robustly assess the estimated canonical correlation coefficients, a permutation test was used. To create the surrogate data, we transformed the original data into the frequency domain, randomized their phases, converted them back to the time domain, and then calculated their magnitudes. The nonnegative canonical correlation coefficients were then estimated with the permuted signals to generate a null distribution. Suppose that this procedure is repeated *N* times, and *M* is the number of permuted results that are larger than the estimated coefficients without permutation; then significance level of the coefficients was estimated as *P*_*val*_=*M*/*N*. In addition, due to the possible collinearity of demographic features, instead of using weights *w* and *v*, the loadings were utilized to represent the contribution of each feature to the canonical variables. The loading is defined as the correlation between each column of *X* and *Y* and the estimated canonical variate.

### 2.7. Linking the Demographic Data with Subnetwork Connectivity Changes

Multiset CCA (mCCA) is a generalization of CCA to multiple datasets by extracting the latent sources from multiple datasets while keeping the correspondence of the sources among the datasets [36]. It optimizes an objective function that includes overall correlations among the canonical variates. For *S* datasets, each has data *X*_*s*_ ∈ *R*^*N∗M*_*s*_^, where *N* is the number of samples and *M*_*s*_ is number of variables, *s*=1,2,…, *S*. The objective function of mCCA can be formulated as

(4) $$\begin{array}{c} \underset{w_{1},w_{2},\ldots w_{S}}{\max}\overset{S}{\underset{i\neq j}{\sum }}w_{i}^{\prime }X_{i}^{\prime }X_{j}w_{j},\\ s.\;t.\;\frac{1}{N}\overset{S}{\underset{i=1}{\sum }}w_{i}^{\prime }X_{i}^{\prime }X_{i}w_{i}\;=1. \end{array}$$

For implementation of the mCCA algorithm, we used the method by Li et al. [36]. We used mCCA to link four datasets together. The first three sets consisted of changes of the interaction coefficients between the three GVS stimuli and baseline state, and the fourth dataset included the demographic data including Unified Parkinson's Disease Rating Scale III (UPDRS III), Montreal Cognitive Assessment (MoCA), Beck's Depression Inventory (BDI), Starkstein Apathy Scale (SAS), Lille Apathy Rating Scale (LARS), Fatigue Severity Scale (FSS), and age.

Significance of the components between the multiple datasets was estimated via the permutation test described above. Permutation tests were performed with 500 iterations and values of *p* < 0.05 were deemed significant. Nonnegative canonical correlation values were first Fisher's *z* transformed to improve their normality.

## 3. Results

### 3.1. Subnetwork Identification

We excluded 5 PD subjects due to failure of the GVS apparatus, which resulted in 15 HC and 27 PD subjects for further analysis.

**Figure 1:**
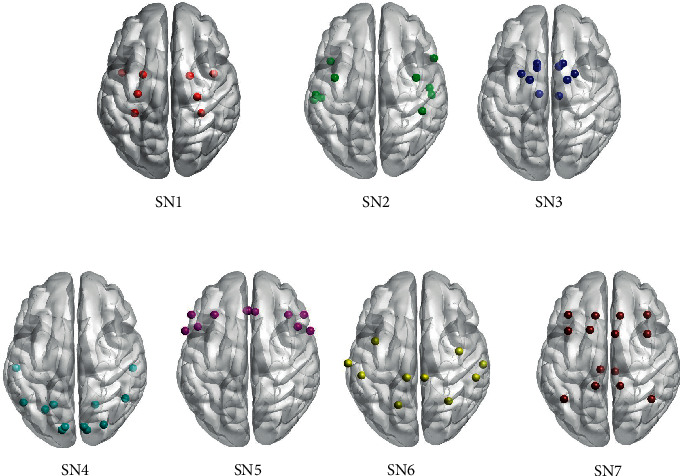
7 subnetworks identified by the modularity maximization and consensus clustering (SN1: hippocampal network; SN2: temporal-insular network; SN3: basal ganglia network; SN4: visual-cerebellar network; SN5: frontal network; SN6: sensorimotor network; SN7: default mode network).

At the subject level, the optimal community structures were identified for each subject and the average number of the modules was seven. To extract the presentative community structures for all subjects across all the conditions (baseline and different GVS stimuli), we applied consensus clustering on the subject-level partitions, and the number of subnetworks was set to be seven according to the average number of modules at subject level. As shown in Figure 1, subnetworks identified were a hippocampal network (SN1), which included the bilateral hippocampal, parahippocampal, amygdala, and entorhinal regions; a temporal-insular network (SN2); a basal ganglia network (SN3), which included the bilateral caudate, putamen, pallidum, accumbens-area, and thalamic regions; a visual-cerebellar network (SN4); a frontal network (SN5); a sensorimotor network (SN6); and a default mode network (SN7), which included the bilateral caudal anterior cingulate, posterior cingulate, precuneus, middle frontal cortex, and inferior parietal cortex regions. The regions included in each subnetwork are listed in Table 4.

To validate the extracted subnetworks based on the connectivity features and compare them with the subnetworks estimated by a traditional approach, we also performed group ICA for all the subjects in all conditions in the common space (Supplementary Figure A1). Thirty group ICA components were estimated, of which at least 17 were considered to be intrinsic networks (a detailed description of the group ICA analysis can be found in Supplementary Appendix B). Six of the seven subnetworks that we detected could be mapped to the 17 components. Notably missing was a basal ganglia network, which is of critical importance for assessment of PD. We therefore restricted our analysis to the ROI-based networks in the native space, as opposed to a group ICA approach in common space.

### 3.2. Subnetwork Interaction Decreases due to PD

**Figure 2:**
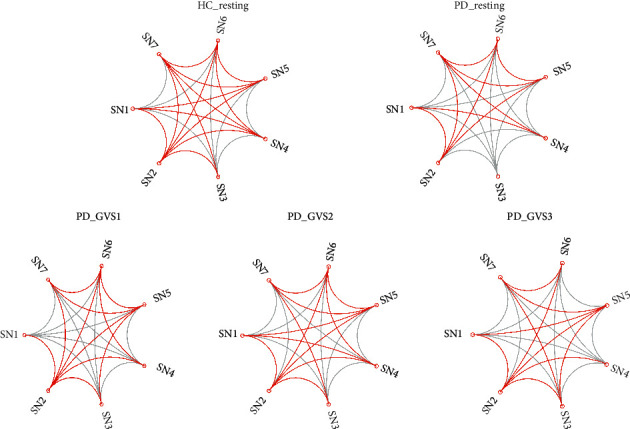
Subnetwork interactions estimated by nonnegative CCA.

**Figure 3:**
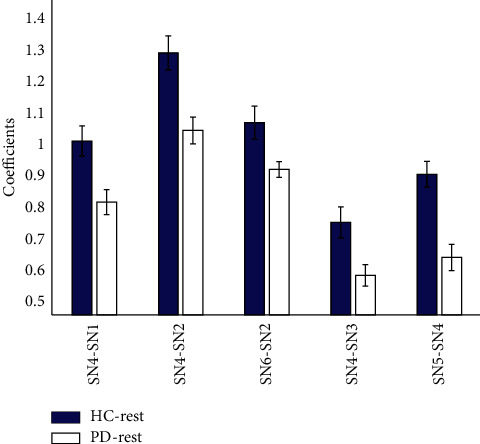
The significant different subnetwork connections between HC and PD group in the resting state. The significance level is <0.05 (false discovery rate corrected).

The subnetwork interactions were estimated by nCCA. As demonstrated in Figure 2, the red lines indicate that the interactions were significant for all subjects. Gray lines indicate that some of the subjects in the group failed to achieve significances. It was noted that PD subjects at the baseline had sparser connections. A two-sample *t*-test was used to compare the subnetwork connections between the HC and PD groups. As shown in Figure 3, the interactions were significantly lower in PD group in the resting state between the visual-cerebellar network (SN4) and the hippocampal (SN1), temporal-insular (SN2), basal ganglia (SN3), and frontal (SN5) networks. Additionally, the connection between the temporal-insular (SN2) network and sensorimotor (SN6) network was also significantly reduced (false discovery rate (FDR) corrected *p* value, pFDR < 0.05).

### 3.3. GVS Improves the Subnetwork Interaction in PD

**Figure 4:**
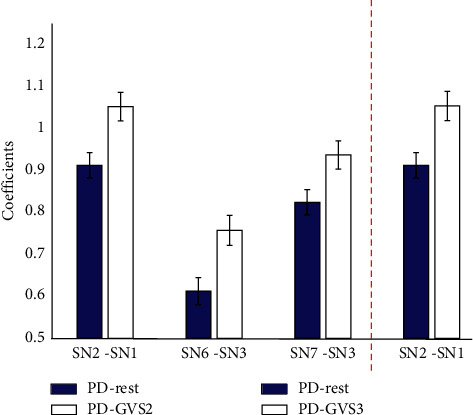
The significant different subnetwork interactions within PD group in the resting state and under GVS stimuli (GVS2 and GVS3). The significance level is< 0.05 (false discovery rate corrected).

To evaluate effects of different GVS stimuli on the subnetwork interactions, we compared the coefficients under GVS with those at the baseline using paired *t*-test. No significant effects of any GVS stimuli on subnetwork interactions were found in the HC group. In contrast, in the PD group (Figure 4), the nCCA coefficients of hippocampal/temporal-insular networks (*p*=0.004, pFDR = 0.0277), basal ganglia/sensorimotor networks (*p*=0.0019, pFDR = 0.0196), and basal ganglia/default mode networks (*p*=0.0002, pFDR = 0.0052) were significantly improved by GVS2 (Figure 4). With GVS3, the interaction between temporal-insular and hippocampal (*p*=0.0006, pFDR = 0.0119) networks was significantly increased. We did not find significant changes in the subnetwork interactions under GVS1.

### 3.4. Demographic Features Associated with the Changes of Subnetwork Interactions under GVS

To examine the associations of demographic data on the changes of subnetwork interactions under GVS, mCCA was utilized to jointly estimate the canonical correlations among four datasets. Three sets represented changes of interaction coefficients between the three GVS stimuli and baseline state, and the fourth dataset included the demographic data including UPDRS III, MoCA, BDI, SAS, LARS, FSS, and age.

**Figure 5:**
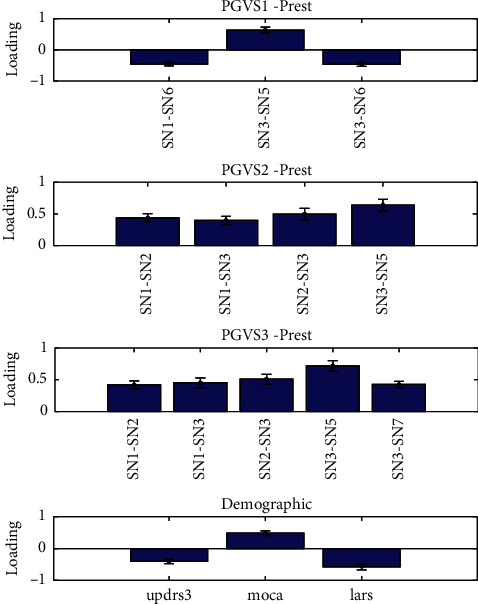
Multiset CCA Component 1. Prest and PGVS represent the PD group in resting state and PD group under GVS, respectively.

**Figure 6:**
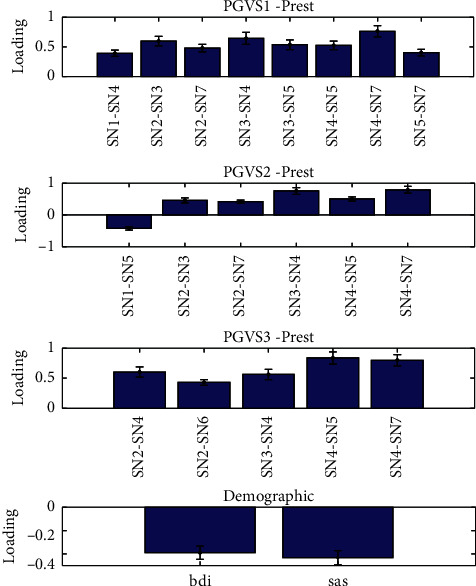
Multiset CCA Component 2. Prest and PGVS represent the PD group in resting state and PD group under GVS, respectively.

**Figure 7:**
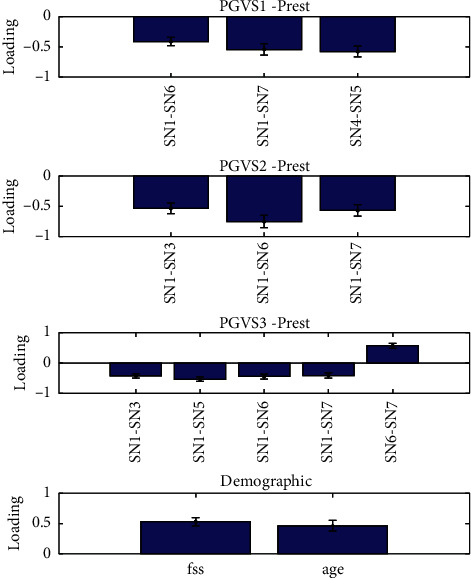
Multiset CCA Component 3. Prest and PGVS represent the PD group in resting state and PD group under GVS, respectively.

Three components were identified (*p* < 0.01) with all pairs of their canonical variables correlated above 0.4. The significance of loadings was assessed by leave-one-subject-out and only the features with significant loadings are reported in Figures 5–7 . For the first component (Figure 5), the UPDRS III, MoCA, and LARS were found to be negatively correlated with a set of subnetwork interaction changes under GVS2 and GVS3. BDI and SAS were found to be related to alterations of subnetwork interactions in the second canonical component (Figure 6). FSS and age had mostly negative correlations with subnetwork interaction changes under three GVS stimuli (Figure 7).

## 4. Discussion

In this paper, we evaluated the effects of different galvanic vestibular stimuli on subnetwork interactions, as neurodegenerative disease is often characterized by connectivity network alterations, particularly those related to small-world network properties [37]. In small-world networks, highly clustered vertex assemblies are connected with a limited number of global shortcuts between clusters, akin to the subnetwork model employed here. When comparing the subnetwork interactions between HC and PD groups in the resting condition (i.e., without stimulation), we found reduced connectivity between the visual-cerebellar network and the hippocampal, temporal-insular, basal ganglia, and frontal networks in the PD group.

The visual-cerebellar network identified in our study was mostly composed of visual-related cortices and cerebellum, which are connected with each other via the cortico-ponto-cerebellar (CPC) pathway [38]. The CPC has been inferred from anatomical and electrophysiological studies in monkeys, which have indicated functional cerebellar connections with visual cortices [39], and resting-state fMRI studies with human subjects have also found functional connectivity between visual cortices and cerebellum [40, 41]. The visual-cerebellar network and other corticopontine projections deriving from the prefrontal cortex, posterior parietal, temporal lobes, and limbic cortices participate in various sensory perceptual processes [38], which are impaired in patients with PD [42]. Cerebellar atrophy in PD (defined by voxel-based morphometry) has been correlated with decreased resting-state connectivity between the cerebellum and sensorimotor, dorsal attention, and default networks while in the on-medication state [43]. Similarly, our findings of impaired connectivity between the visual-cerebellar network and the hippocampal, temporal-insular, basal ganglia, and frontal networks in PD patients on medication indicate that PD is associated with the decoupling among these brain subnetworks, resulting in some PD features such as gait freezing [39]. We note that some studies have suggested that overall functional connectivity of cerebellum is actually enhanced in PD patients off medication, yet it decreased compared to HC when on medication (as was the case here) [44]. The increased cerebellar connectivity in the off-medication state was related to enhanced cognitive and motor performance [44], corresponding to the view of a compensatory cerebellar role to overcome denervation of striatal networks [45]. This suggests that the GVS effects we observed may be consistent with augmentation of compensatory mechanisms.

We applied three types of GVS stimulus including noisy GVS (GVS1), sinusoidal GVS (GVS2), and 70-200 Hz multisine GVS (GVS3) across all participants in order to investigate the effects of different GVS stimuli. When evaluating the subnetwork interactions under different GVS stimuli compared to those at the baseline, no significant differences were found in the HC group. In contrast, in the PD group, sinusoidal GVS and multisine GVS had increased subnetwork interactions for a few pairs of subnetworks (Figure 4). In particular, the subnetwork interaction between the hippocampal subnetwork and temporal-insular subnetwork was significantly improved by both sinusoidal and multisine stimuli. The parietoinsular cortex is a core region for processing vestibular information [46], while the hippocampus is a key anatomical region providing a link between the vestibular system and spatial memory perception [47].

The increase in interaction between the sensorimotor and basal ganglia subnetworks by sinusoidal GVS may be particularly important in PD, where dopamine depletion results in alterations in the basal ganglia-thalamocortical circuits subserving sensorimotor function [48]. When examining the interaction between sensorimotor and basal ganglia subnetwork in the HC and PD groups at baseline, the averaged canonical correlation coefficient was lower in the PD group, so the increase in sensorimotor-basal ganglia interaction by sinusoidal GVS is likely a positive effect for PD. The 1 Hz sinusoidal stimulus we used is approximately at the frequency used for balance research, and, given the increasing recognition of the role of cortical/subcortical interactions in postural control, especially in PD (e.g., [49]), it is unsurprising that this stimulation profile augmented basal ganglia/sensorimotor subnetwork interactions. We note that a prior study investigating fMRI BOLD effects of GVS found maximum effect with 1 and 2 Hz sinusoidal stimuli [50], consistent with the results presented here.

Although beneficial behavior effects of noisy GVS have been suggested in previous studies [6, 51], only sinusoidal and multisine GVS significantly improved subnetwork interactions, while noisy GVS did not (Figure 4). Stochastic facilitation/resonance has been suggested to be a mechanism for some of GVS effects from noisy stimuli [6, 51]. However, stochastic facilitation is usually used to describe effects in an entire system rather than interactions between individual subnetworks. The modest stimulation effects of noisy GVS may be partially attributed to the significant inter- and intrasubject variability, a feature seen in various forms of brain simulation [52]. Several factors may also account for such stimulation variability including anatomical, physiological, and stimulation parameter factors. We suggest that optimization of subnetwork interactions will be an independent dimension upon which to select stimulus parameters.

We further examined the associations between demographic data and changes in subnetwork interactions under different GVS stimuli, in order to relate the GVS effects to cognitive and clinical features. Worsening motor severity and cognition and increased depression, apathy, and fatigue were all negative predictors of most beneficial effects of GVS, suggesting that ultimately patients could be stratified as to their expected responsiveness to GVS stimuli. The *post hoc* analysis adopted in this study is an accessible way to examine the possible factors contributing to the variations of the stimulation effects. However, our study is likely underpowered to determine if subjects should be further stratified by these criteria in future studies.

GVS is a relatively new technique to noninvasively modulate brain activity, and its effect size is still being determined. Most previous studies have had quite small sample sizes, typically less than 20 [11]. In this paper, 32 PD subjects were recruited to validate various GVS experiments. However, 5 PD subjects were excluded due to the failure of the GVS apparatus, resulting in 27 PD subjects in fMRI analysis. While the current study provides further support of the beneficial effects of GVS on PD, continued studies on the full spectrum and magnitude of changes induced by this noninvasive therapy are warranted.

In summary, our results indicate that GVS has widespread system-level influences on brain connectivity patterns and, specifically, can improve subnetwork interactions in PD in a stimulus-dependent manner. The efficacy of such potential therapy is functionally related to demographic variables and disease status. Our study provides a new metric for examining the effects of GVS which could inform future GVS stimulus design.

## Figures and Tables

**Table 1:** Demographic data.

Variables	PD (*n* = 32)	NC (*n* = 15)	*P* values
Gender (female/male)	10/22	5/10	0.838
Age (years)	68.03 ± 5.85	69.4 ± 4.76	0.434
Disease duration (years)	9.16 ± 5.35 [2–26]		
H–Y stage	2.03 ± 0.74 (1-3)		
MDS-UPDRS III (on)	26.5 ± 10.39 [9–51]		
MoCA	25.97 ± 2.53	26.93 ± 1.94	0.1991
BDI	8.53 ± 6.31	3.06 ± 3.90	0.0035
SAS	11.87 ± 5.05	8.07 ± 5.51	0.0236
LARS	-24.25 ± 6.19	-28.8 ± 4.04	0.0128
FSS	3.75 ± 1.60	2.36 ± 1.32	0.0055

**Table 2:** Subscores of the MDS-UPDRS III.

Variables	PD (*n* = 32)
Tremor	3.72 ± 2.30 (0–9)
Rigidity	2.63 ± 2.64 (0–10)
Bradykinesia	10.52 ± 5.31 (0–22)
Gait/posture	3.51 ± 1.70 [1–7]

**Table 3:** 76 ROIs selected in the subnetwork studies.

Index	Name
1	Left-cerebellum-cortex
2	Left-thalamus-proper
3	Left-caudate
4	Left-putamen
5	Left-pallidum
6	Left-hippocampus
7	Left-amygdala
8	Left-accumbens-area
9	Ctx-lh-caudalanteriorcingulate
10	Ctx-lh-caudalmiddlefrontal
11	Ctx-lh-cuneus
12	Ctx-lh-entorhinal
13	Ctx-lh-fusiform
14	Ctx-lh-inferiorparietal
15	Ctx-lh-inferiortemporal
16	Ctx-lh-lateraloccipital
17	Ctx-lh-lateralorbitofrontal
18	Ctx-lh-lingual
19	Ctx-lh-medialorbitofrontal
20	Ctx-lh-middletemporal
21	Ctx-lh-parahippocampal
22	Ctx-lh-paracentral
23	Ctx-lh-parsopercularis
24	Ctx-lh-parsorbitalis
25	Ctx-lh-parstriangularis
26	Ctx-lh-pericalcarine
27	Ctx-lh-postcentral
28	Ctx-lh-posteriorcingulate
29	Ctx-lh-precentral
30	Ctx-lh-precuneus
31	Ctx-lh-rostralanteriorcingulate
32	Ctx-lh-rostralmiddlefrontal
33	Ctx-lh-superiorfrontal
34	Ctx-lh-superiorparietal
35	Ctx-lh-superiortemporal
36	Ctx-lh-supramarginal
37	Ctx-lh-transversetemporal
38	Ctx-lh-insula
39	Right-cerebellum-cortex
40	Right-thalamus-proper
41	Right-caudate
42	Right-putamen
43	Right-pallidum
44	Right-hippocampus
45	Right-amygdala
46	Right-accumbens-area
47	Ctx-rh-caudalanteriorcingulate
48	Ctx-rh-caudalmiddlefrontal
49	Ctx-rh-cuneus
50	Ctx-rh-entorhinal
51	Ctx-rh-fusiform
52	Ctx-rh-inferiorparietal
53	Ctx-rh-inferiortemporal
54	Ctx-rh-lateraloccipital
55	Ctx-rh-lateralorbitofrontal
56	Ctx-rh-lingual
57	Ctx-rh-medialorbitofrontal
58	Ctx-rh-middletemporal
59	Ctx-rh-parahippocampal
60	Ctx-rh-paracentral
61	Ctx-rh-parsopercularis
62	Ctx-rh-parsorbitalis
63	Ctx-rh-parstriangularis
64	Ctx-rh-pericalcarine
65	Ctx-rh-postcentral
66	Ctx-rh-posteriorcingulate
67	Ctx-rh-precentral
68	Ctx-rh-precuneus
69	Ctx-rh-rostralanteriorcingulate
70	Ctx-rh-rostralmiddlefrontal
71	Ctx-rh-superiorfrontal
72	Ctx-rh-superiorparietal
73	Ctx-rh-superiortemporal
74	Ctx-rh-supramarginal
75	Ctx-rh-transversetemporal
76	Ctx-rh-insula

“lh” and “rh” represent left hemisphere and right hemisphere, respectively.

**Table 4:** Seven subnetworks.

Subnetwork index	ROIs
SN1	Left-hippocampus, left-amygdala, ctx-lh-entorhinal, ctx-lh-parahippocampal right-hippocampus, right-amygdala, ctx-rh-entorhinal, ctx-rh-parahippocampal
SN2	ctx-lh-inferiortemporal, ctx-lh-middletemporal, ctx-lh-superiortemporal, ctx-lh-transversetemporal, ctx-lh-insula ctx-rh-inferiortemporal, ctx-rh-middletemporal, ctx-rh-superiortemporal' ctx-rh-transversetemporal, ctx-rh-insula
SN3	Left-thalamus-proper, left-caudate, left-putamen, left-pallidum, left-accumbens-area right-thalamus-proper, right-caudate, right-putamen, right-pallidum, right-accumbens-area
SN4	Left-cerebellum-cortex, ctx-lh-cuneus, ctx-lh-fusiform, ctx-lh-lateraloccipital, ctx-lh-lingual, ctx-lh-pericalcarine right-cerebellum-cortex, ctx-rh-cuneus, ctx-rh-fusiform, ctx-rh-lateraloccipital ctx-rh-lingual, ctx-rh-pericalcarine
SN5	ctx-lh-lateralorbitofrontal, ctx-lh-medialorbitofrontal, ctx-lh-parsopercularis, ctx-lh-parsorbitalis ctx-lh-parstriangularis, ctx-lh-rostralanteriorcingulate ctx-rh-lateralorbitofrontal, ctx-rh-medialorbitofrontal, ctx-rh-parsopercularis, ctx-rh-parsorbitalis ctx-rh-parstriangularis, ctx-rh-rostralanteriorcingulate
SN6	ctx-lh-paracentral, ctx-lh-postcentral, ctx-lh-precentral, ctx-lh-superiorparietal, ctx-lh-supramarginal ctx-rh-paracentral, ctx-rh-postcentral, ctx-rh-precentral, ctx-rh-superiorparietal, ctx-rh-supramarginal
SN7	ctx-lh-caudalanteriorcingulate, ctx-lh-caudalmiddlefrontal, ctx-lh-inferiorparietal ctx-lh-posteriorcingulate, ctx-lh-precuneus, ctx-lh-rostralmiddlefrontal, ctx-lh-superiorfrontal ctx-rh-caudalanteriorcingulate, ctx-rh-caudalmiddlefrontal, ctx-rh-inferiorparietal ctx-rh-posteriorcingulate, ctx-rh-precuneus, ctx-rh-rostralmiddlefrontal, ctx-rh-superiorfrontal

## Data Availability

Imaging data were collected from Pacific Parkinson's Research Center (PPRC) at the University of British Columbia (UBC).

## Supplementary Materials

Supplementary Materials In Supplementary Appendix A, the consensus clustering is introduced to estimate the representative community structure by combining partitions of all the subjects. To validate the extracted subnetworks based on the connectivity features, the subnetworks derived from group ICA were examined in Supplementary Appendix B.
