# Supplementary material for: Galvanic Vestibular Stimulation Improves Subnetwork Interactions in Parkinson's Disease
Source: J Healthc Eng. 2021 May 13;2021:6632394. doi: 10.1155/2021/6632394 (PMC8137296; doi:10.1155/2021/6632394)
Supplement: Supplementary Materials — In Supplementary Appendix A, the consensus clustering is introduced to estimate the representative community structure by combining partitions of all the subjects. To validate the extracted subnetworks based on the connectivity features, the subnetworks derived from group ICA were examined in Supplementary Appendix B. [file 6632394.f1.docx]

# Supplementary

### Appendix A: Consensus clustering

Consensus clustering has been widely adopted to estimate common community structures by combining multiple partitions together, leading to a robust representation of the inner structure and integrating the information from distributed sources [[1](#_ENREF_1)]. It offers a natural solution to detect group partitions from the individual subject level clustering. Suppose $X=(x_{1},x_{2},\ldots\ldots,x_{N})$ represents $N$ ROIs (variables). The partitions for $M$ subjects are $\Pi=\{\pi_{1},\pi_{2},\ldots\ldots,\pi_{m}\}$ and each $\pi_{i}$ for subject $i$ is the mapping from $X$ to $\{1,2,\ldots\ldots,K\}$ where $K$ is the number of clusters in $\pi_{i}$. A cluster based similarity between each pair of ROIs can be defined as,

$$S_{ij}=\frac{1}{M}\sum_{m=1}^{M} I(\pi_{m}\left( x_{i} \right)=\pi_{m}(x_{j}) )$$

where $I$ is indicator function [[2](#_ENREF_2)]. Therefore, we generated a similarity matrix between ROIs by the co-occurrence of ROIs in the same clusters among all the partitions, leading to a new set of similarity relationships, and the modularity maximization at the group level was further applied to obtain the group representative subnetwork structures.

### Appendix B: Group ICA

#### Preprocessing in the common space

#### To preprocess the collected fMRI data in the common space, DPABI (<http://rfmri.org/dpabi>) and SPM8 software package (<http://www.fil.ion.ucl.ac.uk/spm>) were utilized. The first 5 time points were discarded to allow the magnetization approaching a dynamic equilibrium and the remaining images were corrected for slice timing as well as motions for any major head movements during the scan. Co-registration was performed between functional data and structural images. Imaging data were then normalized into the standard Montreal Neurological Institute template, and resampled to 3.0 × 3.0 × 3.0 mm isotropic voxels. In the next step, fMRI signal was detrended by removing any linear or quadratic trends. Nuisance time courses were then voxel-wise regressed from the processed data to remove potential confounds of head motion with Friston-24 correction. The fMRI data were finally spatially smoothed by a 6×6×6 FWHM Gaussian kernel and bandpass filtered at 0.01 Hz to 0.08 Hz.

#### Group ICA

Independent component analysis (ICA) is a blind source separation approach that has been increasingly utilized for assessing resting state fMRI data by decomposing the BOLD signals into spatially independent maps and their associated time courses as [[3](#_ENREF_3)],

$$X=AS$$

where $X\in R^{T*N}$ denotes the observations (i.e. fMRI voxel-wise time courses). The sample length is $T$ and the number of voxels is $N$. $S\in R^{L*N}$ is the underlying sources with each row representing one independent spatial map. $A$ is the mixing matrix.

To further make inference about a population rather than focusing on an individual subject, the group ICA has been proposed to obtain the common spatial maps and unique time courses for all the subjects by temporally concatenation of multiple fMRI data sets [[4](#_ENREF_4)]. Prior to performing group ICA, two levels of dimension reduction of fMRI were conducted by Principal Component Analysis (PCA). At the subject level, fMRI date was firstly reduced to 45 PC components and all the PC components were concatenated for the subsequent analysis. The number of group level PCs was set to be 30 and 30 group ICA components were estimated. The group ICA was implemented by GIFT Matlab toolbox (<http://icatb.sourceforge.net/>).

As demonstrated in Figure B1, 17 of 30 components were considered to be the intrinsic networks. However, a basal ganglia network, of vital importance for assessment of PD, was not identified. Therefore, in this study, we restricted our analysis to the ROI-based networks in the native space, as opposed to a group ICA approach in common space.

### Reference

1. Lancichinetti, A. and S. Fortunato, *Consensus clustering in complex networks.* Scientific Reports, 2012. **2**: p. 336.

2. Nguyen, N. and R. Caruana. *Consensus clusterings*. in *Data Mining, 2007. ICDM 2007. Seventh IEEE International Conference on*. 2007. IEEE.

3. McKeown, M.J., et al., *Analysis of fMRI data by blind separation into independent spatial components.* Human brain mapping, 1998. **6**(3): p. 160-188.

4. Calhoun, V.D., J. Liu, and T. Adalı, *A review of group ICA for fMRI data and ICA for joint inference of imaging, genetic, and ERP data.* Neuroimage, 2009. **45**(1): p. S163-S172.

## Figure legends


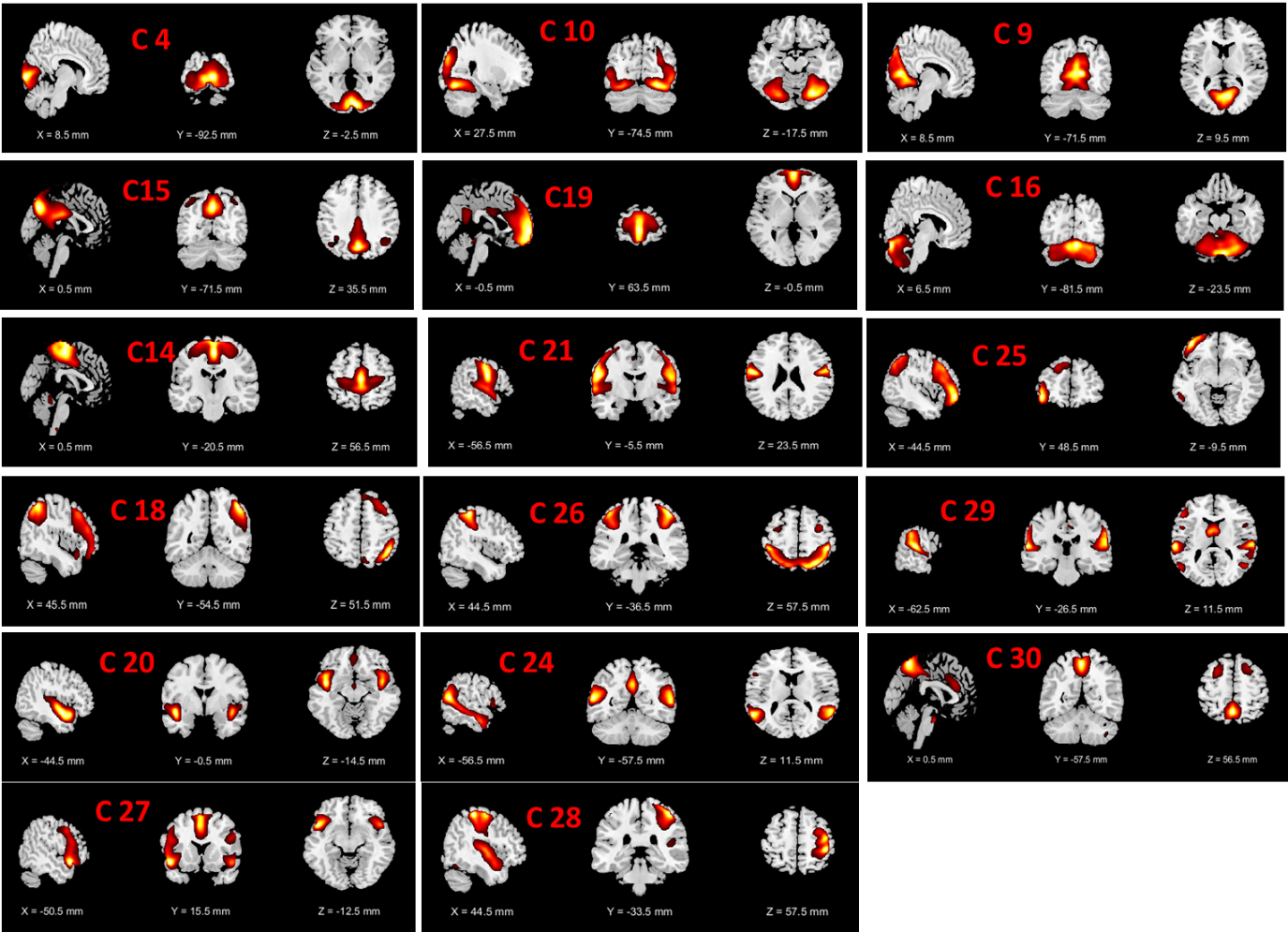


Figure A1. Independent components estimated by group ICA.
